# Coordination of Family Caregiver Support Services Across VHA

**DOI:** 10.1093/geroni/igaf122.322

**Published:** 2025-12-31

**Authors:** Emma Quach, Ryan Cabrera, Victoria Ngo, David Giard

**Affiliations:** VA Bedford Health Care System, Bedford, Massachusetts, United States; VA Bedford Health Care System, Bedford, Massachusetts, United States; VA Bedford Health Care System, Bedford, Massachusetts, United States; Department of Veterans Affairs, Jamaica Plain, Massachusetts, United States

## Abstract

Veterans Affairs (VA) primary care (PC) currently treats more Veterans with dementia than any other VA setting, due to a scarcity of geriatricians and neurologists. In the course of caring for Veterans with dementia, frontline PC nurses frequently interact with family caregivers, whose support enables Veterans with dementia to live where they want—in their homes—but such support may lead to caregiver burnout, contributing to Veterans requiring institutional care. Screening for and referral to caregiver support services in PC may prevent or reduce caregiver burnout but are reportedly rare in non-VA settings. To explore caregiver screening and support by VA PC, we conducted an online focus group with 23 nurses working at a New England VA medical center. Qualitative analysis showed PC nurses do not formally conduct screening. They, however, recognized addressing caregiver burnout to be part of good patient care and were acutely concerned about the varied dilemmas facing caregivers and Veterans. For example, nurses struggle to work with family caregivers’ stress related to their need to balance Veterans’ independence and safety. To support distressed caregivers, nurses valued easily accessible information on VA caregiver support groups, caregiver self-care, and VA respite service eligibility criteria (services administered outside of PC) and consults to PC social work. Nurses’ reflections revealed consistent coordination among these VA entities is essential to provide seamless support to family caregivers seen with Veterans in PC. Our study suggests the integral role of PC nurses in supporting family caregivers as part of providing holistic care.

